# The Influence of Genetic and Environmental Factors and Their Interactions on Immune Response to Helminth Infections

**DOI:** 10.3389/fimmu.2022.869163

**Published:** 2022-04-29

**Authors:** Oyebola O. Oyesola, Camila Oliveira Silva Souza, P’ng Loke

**Affiliations:** Laboratory of Parasitic Disease, National Institute of Allergy and Infectious Disease (NIAID), National Institute of Health, Bethesda, MD, United States

**Keywords:** genetics, environment, interaction, Helminth infection, heterogeneity

## Abstract

Helminth infection currently affect over 2 billion people worldwide, with those with the most pathologies and morbidities, living in regions with unequal and disproportionate access to effective healthcare solutions. Host genetics and environmental factors play critical roles in modulating and regulating immune responses following exposure to various pathogens and insults. However, the interplay of environment and genetic factors in influencing who gets infected and the establishment, persistence, and clearance of helminth parasites remains unclear. Inbred strains of mice have long been used to investigate the role of host genetic factors on pathogenesis and resistance to helminth infection in a laboratory setting. This review will discuss the use of ecological and environmental mouse models to study helminth infections and how this could be used in combination with host genetic variation to explore the relative contribution of these factors in influencing immune response to helminth infections. Improved understanding of interactions between genetics and the environment to helminth immune responses would be important for efforts to identify and develop new prophylactic and therapeutic options for the management of helminth infections and their pathogenesis.

## Introduction

Research into factors that influence host response during helminth infection are usually focused on use of inbred mice raised in a specific pathogen free environment as found in most research institutes and academic institutions across the world. However, in the real-world setting, helminth infections are characterized by infections of individuals living in various communities with different lifestyles as well as with wide genetic variations. Also, the intensity of helminth infection among individuals varies markedly and can be influenced by various genetic and environmental factors (1, 2). Therefore, in this review, we discuss the influence of genetic and environmental diversities in the regulation of helminth induced immune response and their contribution to inter-individual variation seen in responses during helminth infection (3, 4). Furthermore, we highlight ongoing studies and future opportunities to examine the interaction between environment, genetics and other variables that influences the interindividual variation seen during helminth infection.

## Immune Response to Helminth Infection

Studies with genetically modified mice on the C57BL/6 background has transformed our understanding of Type 2 responses to helminths in the last few decades. Immune responses during helminth infection are characterized by recruitment and accumulation of innate immune cells such as eosinophils, basophils, innate lymphoid cells (ILC2), neutrophils, alternatively activated macrophages as well as cell of the adaptive immune system such as B cells, Th2 and T regulatory CD4 T cells (5–11). These cells produce Type 2 and regulatory cytokines and other mediators which play important protective and regulatory functions during helminth induced inflammation (12–14). Recent advances demonstrate the critical role other previously overlooked cells such as epithelial, neuronal, and stromal cells play in contributing to and regulating Type 2 immune responses during helminth infection (5, 15, 16). These non-immune cells can produce cytokines, alarmins and other bioactive mediators that crosstalk with innate and adaptive immune cells to regulate the response during helminth infection. Despite these advances, inter-individual variation in these responses and the influence of the environmental interaction with host genetics in free-living mammals is not well understood.

## Genetic Variation in Resistance to Helminth Infection

Our ability to genetically manipulate mice has been fundamental for increased understanding of mammalian physiology. As this technology evolved over time, the C57BL/6 strain of mice has emerged as the inbred strain of choice for most immunological studies. As a result, our understanding of the basic mechanisms that surround immune response during helminth infection has also improved significantly with the use of genetically modified mouse models for dissecting various mediators and immune cell populations in the regulation of helminth induced immune responses. Increasing complexity of mouse models include cell specific knockouts, genetic inducible fate mapping models, in addition to the global knock-out and/or transgenic mice models have been critical in identifying new pathways that regulate Type 2 and immunoregulatory responses to helminths. To reduce variation in these reductionist experiments to characterize detailed mechanisms, genetically identical mice strains of similar age groups and sometime sex are used to isolate and study the role of specific cell types and immune mediators.

However, various other studies have used in-bred strains of mice to study the role of genetic variations in resistance to helminth infection (17). For example, previous studies have shown that the BALB/c strain of mice are more susceptible to *Litomosoides sigmodontis*, a filarial nematode, compared to the C57BL/6 mice or the C57BL/10 mice (18–20). This contrasts with other intestinal helminth parasites such as *Trichuris muris* (21–23) and *Heligosomoides polygrus* (24–26) where the BALB/c strain has been shown to be more resistant. Other studies have also examined inbred strains with various other helminth parasites (**Table 1**). While genetic variation clearly influences susceptibility and resistance to helminth infection in mouse models, our understanding of the basic mechanisms elicited during helminth immune response critical for mediating these differences remain unclear. While mechanisms driving these differences include the role of various effector immune cells, cytokines, and immunoglobulins (**Table 1**), the role of genetic variation in regulating primary and/or secondary sentinels of Type 2 inflammation (55–57), such as epithelial, stromal, and neuronal cells is currently less appreciated and has not been well studied. For example, in the area of epithelial cell biology, C57BL/6 and BALB/c mice show differences in tuft cell response at steady state and in response to a protozoa parasite, *Trichomonas muris*, but no significant difference was seen in tuft cell response following chronic infection with *H. polygyrus* at peak of parasite establishment (58). The dynamics of tuft cell hyperplasia in the different inbred strain of mice could vary wherein the BALB/c mice might have higher response than the C57BL/6 mice (59). Hence, the role of these sentinels in the pathogenesis and outcome to helminth infection should be examined in different inbred strains of mice.

**Table 1:** Understanding the role of naturally occurring genetic variation in resistance and outcomes to helminth infections – mice models.

Genetic Susceptible strains	Helminth infection	Type of Helminth Parasite	Protective/Susceptible mechanistic explanation	References
BALB/c	*Litomosoides sigmodontis*	Filarial Parasites	CD4 T lymphocytes; production of IL-4	(18–20, 27)
AKR; B10.BR	*Trichuris muris*	Whipworm	Higher Th1 effector response characterized by increased IFN gamma production	(21–23, 28–31)
CBA; C3H; SLA/J; C57BL/6; C57BL/10	*Heligmosomoides polygyrus*	Hookworm	Decreased Th2 driven effector response characterized by lower IgE responses, lower intestinal mast cell densities, alternatively activated macrophages and a concomitant increase in TNFα and IFN γ response; Increased proportion of CD103+FoxP3+ activated T Regulatory cells in susceptible strains;	(24–26, 32–37)
C57BL/6	*Ascaris suum*	Round worms	Hepatic factor, less intense inflammatory and repair response in the liver? Role of secretory IgA	(38–40)
CBA; BALB/c; C57BL/6;	*Nippostrongylus brasiliensis*	Round worms	Developmental arrest in the lungs or migration deficiency of larva into the intestinal tissue in resistant mice, FVB/N; Immunological mechanism is not clear, possibly a Type 2 dependent immune response that limits tissue associated immune response	(41–43)
BALB/c, DBA/2	*Taenia crassiceps*	Tape worms	T cell dependent mechanism. Role of Regulatory T cells	(44–48)
C57BL/6	*Trichinella spiralis*	Round worms	Mucosal mast cells	(49)
CBA; C57BL/10	*Schistosoma mansoni*	Blood flukes (trematodes)	Increased IL-1β and IL-23 cytokines by DCs and T helper 17 polarization; Proinflammatory T helper 1/T helper 17 responses persist along with T helper 2; Reduction of the alternative activation marker	(50–54)

Studies in other free-living mammals such as the livestock population and wild animals has also highlighted the importance of genetic factors in susceptibility to helminth infection (60–64). For example, farmers and livestock breeders will often use their knowledge of breed specific resistance and susceptibility to helminth infection to minimize cost and losses associated with infection with helminths, by selecting helminth parasite resistant strains for breeding. Some studies have linked these genetic resistant and susceptibility patterns to the protective Type 2 mechanism (60, 64, 65).

There are fewer studies in the human population that provides a mechanistic understanding to the influence of genetic variations on susceptibility to helminth infection, despite substantial evidence for the role of genetics in determining susceptibility to infections (1). Logistical and ethical constraints often limit human population studies to correlational observations rather than a study of cause-and-effect relationships. Currently, host genetics is said to account for about 20 to 40% of variation in intensity of worm burdens seen during helminth infections (1). For example, a few studies have demonstrated the role of genetic factors in susceptibility to human Ascaris infection (66–68). Notably, they were able to associate this genetic factor to a peak in chromosome 13 which is close to the known locus of a major candidate gene, *TNFSF13B*, involved in the regulation of B cell activation and immunoglobulin secretion (68–71). A few other genetic factors such as during *Trichuris trichiura* infections have also been identified (72) and associated with localization of two significant quantitative trait loci on chromosomes 9 and 18, which contains genes that can influence immunoregulatory cytokines like IL-10 (73). Susceptibility to other helminth parasites such as blood flukes like *Schistosoma mansoni* and hookworms like *Necator americanus* and *Ancylostoma duodenale* has also been linked to genetic factors (74–78). What is unclear is when linkage of susceptibility to helminth infection involves more than one genetic locus, whether this is dependent on one gene or if genetic variation in other genes could affects resistance to helminth parasite. Perhaps mechanistic insights could be gleaned from mouse models, if these regions are conserved, to understand the relationship and function of those genes. Therefore, such clinical and genetic epidemiology studies may guide the conduct of fundamental and basic immunology experiments.

Host genetics could also indirectly influence other host associated factors which then indirectly influence the immune system. For example, difference in expression of major histocompatibility complex molecules can influence the composition of the gut microbiome (79–82) through differences in antibody responses against commensal bacteria (80). Incidentally, these MHC associated differences in gut microbiome may then influence subsequent susceptibility to helminth infection (79). Hence, genetic factors may alter the host microenvironment to affect subsequent outcomes to helminth infections. However, because these are observational studies in primates, it is challenging to mechanistically isolate the effect of immune mechanisms resulting from different MHC haplotype and effects due to microbiome differences.

## Genetic Variation in Pathogenesis of Helminth Infection

It is important to distinguish between the role of genetic variation in resistance and pathogenesis to helminth infections. Although resistance and pathogenesis are linked because disease morbidity is often observed in heavily infected individuals, mechanisms that drive disease pathogenesis may be unrelated to mechanisms responsible for parasite resistance. Our understanding of pathogenesis during helminth infections is based primarily on studies with genetically modified mouse models, which provide insights into the pathways, cytokines and other mediators that regulate the disease processes. Also, differences in disease outcomes from infection of inbred strains of mice have provided additional clues in the heterogeneity of immune response and severe pathology.

The cytokine balance during infection is an important determinant of pathogenesis, mediating both resistance and tolerance to infection. Rapidly after infection, pathogen associated molecular patterns are detected by pattern recognition receptors (PRRs) together with release of alarmins like IL-33, IL-25 and TSLP at epithelial barriers (83, 84). This leads to activation of transcription factors, such as STAT6 and GATA3, which subsequently induce the upregulation of sets of genes including receptors, cytokines, chemokines, and genes regulating the production of eicosanoids (85–87). Cytokines, chemokines, and eicosanoids can induce recruitment, accumulation, and differentiation of immune cells with release of additional sets of effectors cytokines that then induce repair, differentiation, and release of effector molecules from the epithelial barrier to help in the clearance of the worms (87–90). Thus, cytokines, chemokines, eicosanoids, and other mediators are key in the initiation as well as in the pathogenesis of anti-helminth immune responses.

Alterations to the balance of the cytokine response because of host genetic variation can affect the inflammatory mediator profile which can determine the pathogenesis of helminth infection. For example, C57BL/6 and CBA mice show different levels of immunopathology during *S. mansoni* infection, when challenged with a similar number of cercariae. This may be due to differences in the switch from a pro-inflammatory T helper 1 (Th1)/T helper 17 (Th17) response to a tissue protective Type 2 response. The CBA mice show higher levels of proinflammatory Th1/Th17 cytokines which does not diminish but instead persists alongside the rising T helper 2 (Th2) responses, while C57BL/6 mice can regulate the Th17 response during the expansion of Th2 cells resulting in milder pathology (50–52). However, it is notable that a prolonged Th2 response may lead to other forms of pathology such as increased fibrosis in the BALB/c mice (91). Similarly, *S. mansoni* infection in humans can have different outcomes and pathophysiology, ranging from the mild symptoms to severe symptoms such as the development of severe hepatic fibrosis, hepatosplenic disease, ascites, and encephalopathy, irrespective of the intensity of infection (77, 78). In some cases, a genetic explanation has being proposed in individuals with severe pathological outcomes with some evidence of polymorphisms in cytokine and cytokine receptor genes like *IFN-γR1* gene, which encodes the receptor for the Type 1 cytokine, IFN-γ (78, 92), those involving *TGFBR2* which encodes for receptor of regulatory cytokine, TGF-β (78) and *IL-22RA2*, which encode the receptor for IL-22 (93). Other genes such as polymorphisms in *CNN2* gene, which encodes for the connective tissue growth factor (CTGF), a stromal factor, has also been implicated in the pathophysiology of these disease (94, 95).

Resistance to helminth infection in host of different genetic backgrounds may also be associated with roles of different cytokines in regulating the outcome to infection. For example, differences in susceptibility to *T. muris* infection in C57BL/6 mice compared to BALB/c mice might be due to the role of different cytokines with IL-4 playing a predominant role in C57BL/6 mice and IL-13 playing a more predominant role in BALB/c mice (96). In addition, the protective immunity in C57BL/6 mice vaccinated with radiation-attenuated (RA) larvae of *S. mansoni* is associated with Th1 immune response, while on BALB/c background the protection depends on Th2 responses. Here, injection of RA-attenuated larvae produced lower levels of IgG1 antibodies in serum IL-4Rα deficient mice (IL-4Rα^-/-^) on BALB/c background, but the serum from vaccinated wild-type BALB/c mice confers protection to IL-4Rα^-/-^ mice, suggesting the Th2 antibodies is crucial for parasite elimination and resistance in BALB/c mice (97).Genetic differences due to a distinct pattern of cytokines secreted and markers expressed by myeloid cells can also correlate with different helminth infection outcomes. For example, dendritic cells (DCs) from *S. mansoni-*infected high morbidity CBA mice display increased expression of CD209a (C-type lectin receptor – CLRs) which is necessary for the production of IL-1β and IL-23 that drives pathogenic Th17 cells, as opposed to the low morbidity C57BL/6 mice (51, 52, 98). Similarly, C57BL/10 mice develop more severe schistosomiasis, as defined by significant larger granulomas, increased proinflammatory cytokines production by DCs and higher levels of IL-17 compared to C57BL/6 mice. This phenotype is tightly connected to DCs function, because DCs from C57BL/6 mice expressed high levels of Ym1 and RELMα, marker of alternative activation that regulates the tissue repair in responses to *S. mansoni* eggs (53).

Population genetics studies in humans have shown that the number of pathogens in a specific geographic region can have selective pressure on genes related to cytokine production and responsiveness (99–101), regulation of cellular responses (102) as well as transcription factors important for the induction of protective Type 2 immune response during helminth infection (103, 104). This suggest that evolutionary pressure in different geographic location with different parasite profiles can influence polymorphism of genes and regulatory elements that can then affects response to parasites endemic in those regions.

### Parasite Factors as Source of Variation to Host Responses During Helminth Infection

In addition to host genetic factors, variation in parasite species and genetics can also be a major contributor to heterogeneity in susceptibility and resistance patterns during helminth infection (105–108). For instance, variation in reproductive output and consequently egg production following helminth infection has been attributed to parasite genetic factors (108). Parasite genetics could also influence the properties of excretory and secretory proteins released from the worms, which could in turn influence host-parasite interactions as well as parasite chronicity patterns (109, 110), the ability of the parasite to evade the host immune system (110) or the pathogenesis of immune response in the host (107, 108, 111–114). For example, differences in lifecycles and egg properties between *S. haematobium* and *S. japonicum* results in varied cellular and humoral immune responses despite belonging to the same helminth parasite genus (108, 111, 112).

Moreover, different stages of the lifecycle are also an important source of heterogeneity in host responses. As seen in *S. mansoni*, there is a clear shift from a Th1 mediated immune response to a Th2 immune response at the onset of egg production (115), while the cercariae and larva induce Th1 dominated immune response (116). The different lifecycle stages also produce different suites of excretory/secretory products that modulate the host response in diverse ways to promote invasion, infection, adhesion and the immunoregulatory process (108, 117, 118). Hence, parasite factors are a critical source of heterogeneity in immune responses during helminth infection.

Furthermore, in mouse models of infection, strain specific differences have also been described. Strains of *T. muris* isolated and maintained in different parts of the world, including the S strain (isolated in Sobreda, Portugal), the E strain (isolated in Edinburgh) and the E-J strain (originally E strain, which has been maintained in Japan since 1969) can induce different immune response following infection of mice. The E and E-J strains of *T. muris* generally induce a Th2 skewed response whereas the S-strain induces a Th1 skewed response characterized by production of IFN-gamma and IL-12 (107, 113, 114). Therefore, heterogeneity in parasite genetics can be a source of variation in immune response and perhaps helminth parasites can adapt their metabolism and alter the immune response to fit into their environment (109, 119). Since most laboratory strains have been maintained and passaged in different laboratories for several years, it is possible that parasites used in most laboratories diverge significantly from wild helminth parasites that are present in their natural environment.

## Role of Environment During Helminth Induced Type 2 Immune Response

There are many environmental variables that can affect parasite burden in an endemic population and dissecting the relative contribution of each variable can be difficult. Most helminth infections are soil transmitted parasites; thus, the host environment is important in determining exposure to and transmission of helminth parasites (2). In addition, the environment also plays a crucial role in determining variation in immune responses, pathogenesis of diseases as well as susceptibility versus resistance patterns during helminth infection. This can be the biotic environment of the individual which can constitutes the commensal microbial communities within the host or the other free living organismal life the host interacts within the environment including vectors and intermediate host for parasites. Other biotic factors including previous microbial experience and infection history of the host are important in influencing susceptibility and resistance to helminth infection (120–122). For example, primary infection activates memory CD4^+^ T cells and alternatively activated macrophages that mediate resistance against secondary helminth infection (43, 123–125). There are also examples whereby previous helminth infection can make the host more susceptible to secondary helminth infection (126). Indeed, helminth co-infection may influence outcome of other infections and many studies have investigated the influence of previous helminth infections on the pathogenesis of other infectious diseases – including bacterial, protozoan, and viral diseases (120–122, 127–134). This experimental study design stems from the idea that mammals have co-evolved with helminth parasites and they could be part of the natural macrobiota of the host (135, 136). However, pre-existing disease and co-infections may also influence outcomes during helminth infection. For example, prior infection and co-infection of the protozoan parasite *Toxoplasma gondii* with the enteric nematode *H. polygyrus* can limit the host protective Type 2 immune response directed against the helminths making the host more susceptible to the worms (137). This results from the immune landscape in the host being skewed towards a Type 1 response by the protozoan parasite (137). A similar phenomenon has been reported wherein prior infection with protozoan parasites like *T. gondii* and *Plasmodium* parasites and viral pathogens like Human T-lymphotropic virus 1 (HTLV-1), which all induce a Type 1 immune response, can limit the host response during subsequent infection with helminth parasites like *Fasciola hepatica*, *Nippostrongylus brasiliensis* and *Strongyloides stercoralis* (122, 138–141). Hence, prior and co-infections by other pathogens can influence pathogenesis and susceptibility to helminths, thereby potentially contributing to inter-individual variations in immune response during helminth infection. In addition to biotic factors, these could also involve abiotic factors which constitute climatic factors such as the temperature, humidity, rainfall; physical factors such as the soil and mineral composition of the environment; and chemical factors such as the oxygen, nitrogen, and CO_2_ levels in the environment. Factors such as this can influence the lifecycle of the parasite outside the host and therefore transmissibility of the helminth parasite. These biotic and abiotic factors can also have major implications on the tone of immune response as well as host susceptibility to helminth parasite infection thereby contributing to inter-individual variation during helminth infection (142).

**Figure 1:**
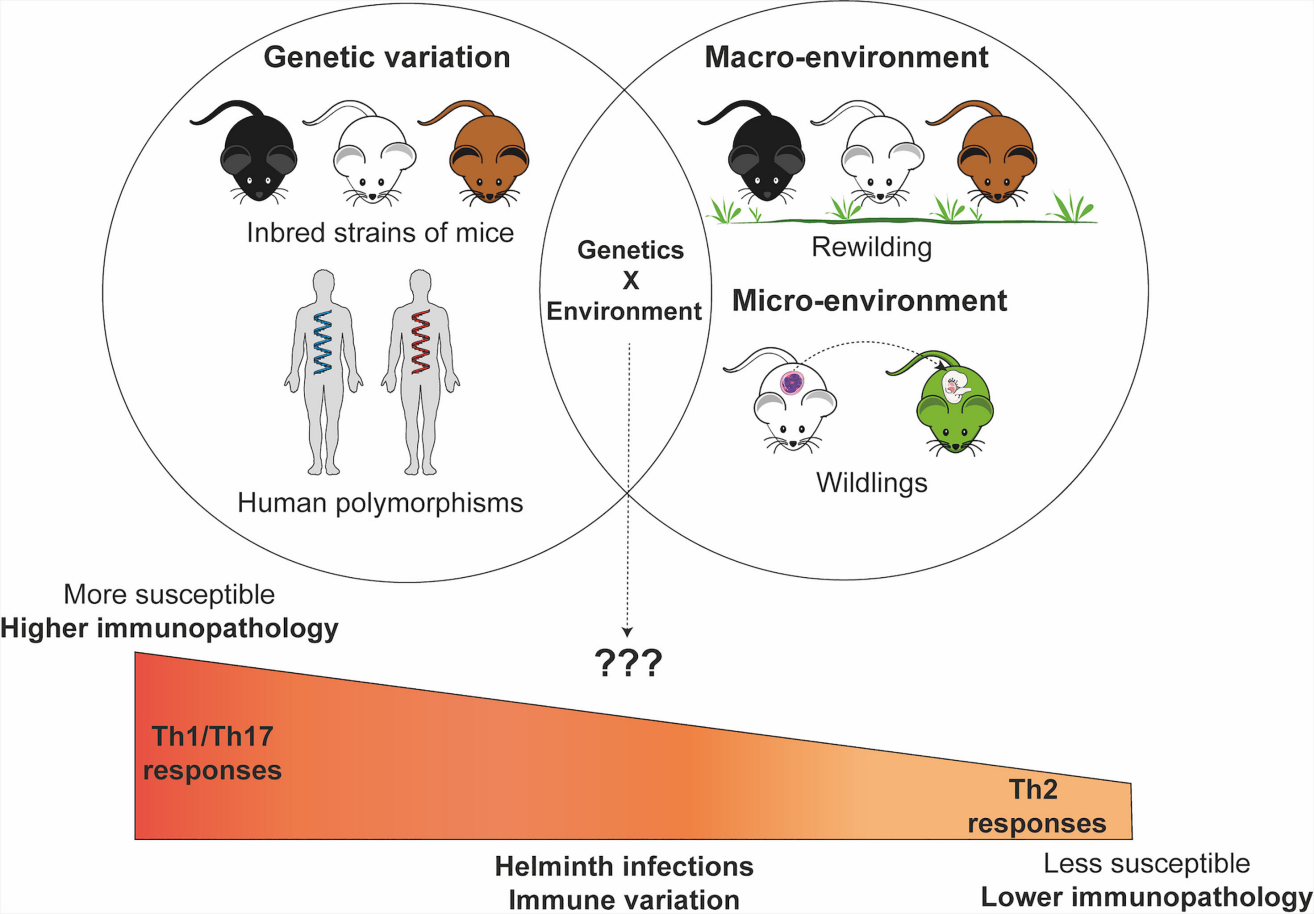
Genetic and environmental factors contribute and interact to influence susceptibility patterns and pathogenesis of immune response during helminth infections. The Figure in this manuscript was created using images from the Servier Medical Art’s image collection (smart.servier.com) and licensed under a Creative Commons Attribution 3.0.

Several studies have shown that the environmental differences can significantly influence the host immune phenotype and profile (143–146) and consequently susceptibility to subsequent helminth infections (147) (**Figure 1**). The importance of the role of environmental influences on the immune system can be appreciated from twin studies which show that variability in immune responses can be dictated in large part by acquired and not only genetic factors especially with increasing age emphasizing the influence of environmental factors on immune responses (148). Although, similar twin studies experiment in the context of helminth infection have not really been done in humans to understand the role of environment versus genetics during helminth infection. The use of mice models such as the rewilding mice model has helped us appreciate the critical role the host environment can play in outcomes during *T. muris* infection (147, 149). However, there is still a need to understand how the tissue microenvironment (3) and/or host macroenvironment (142, 150) can influence susceptibility patterns and helminth infection outcomes during exposure to various other helminth parasite types (hookworms, roundworms, tapeworms). New environmental mouse models such as “rewildings”, “wildlings”, “co-housing”, “sequential infection models”, “co-infection models”, “chimeric wild mouse” etc (142, 146, 151–153) provide new opportunities to understand the role of environmental factors in influencing susceptibility to infection and re-infection by helminth parasite.

Rewilding mice involves introducing laboratory mice into an outdoor enclosure. This outdoor enclosure exposes mice to a natural environment including soil, weather, vegetation, microbial population, but protects against predation, and serves as a bridge between laboratory controlled experiments and what happens in a more natural environment (147). The rewilding mice model has already provided critical insights into the role of host macroenviroment on helminth infection outcomes (147, 149). Rewilded mice were more susceptible to the intestinal helminth parasite *T. muris* with higher worm burdens as well as more worm biomass than the laboratory controls (147). Rewilding experiments have also revealed how the environment contributes to the microbial diversity in the gut (149), provided insights into the role of fungal colonization to neutrophil circulation (143) as well as uncovered the role of environment and genetic factors in immune composition and responsiveness (144).

Besides rewilding, other approaches focus on increasing the microbial diversity in the gut environment, including fecal transplants, co-housing, exposure to dirty bedding (formites) and embryo transfer from wild and pet store mice (145, 146, 151, 153, 154). Thereby leading to various model including “wildlings”, “chimeric lab-wild models”, which have a natural and diverse metaorganisms at all body sites similar to wild and petstore mice (145, 146). These mice may have better translational value than specific pathogen free laboratory mice found in most biomedical centers with immune systems that are a better reflection of the human situation (142, 146, 152, 153). These mice mount an immune response that limits exuberant responses which promotes survival following intense inflammatory conditions and are better able to control pathogen exposure compared to their SPF counterparts (145, 151). However in some cases, these mice generate stronger immune responses than their controls, for example, during exposure to house dust mite antigen (155). Other models have focused on sequentially infecting, co-infecting, persistently infecting SPF mice with various known microbial pathogens to expand their microbial experience, immunological landscape and microenvironment so that they better reflect the human experience (129, 156). This delivers a controlled set of pathogens to the mice and eliminates the requirement for special housing facility in order to use these mice to test various hypotheses in biomedical research.

In conclusion, there is considerable interest in developing these new tools (142, 152) to better model the human immune response in mice and to assess the role of environment and its interaction with genetic factors on susceptibility and resistance patterns during helminth infection.

### The Microbiome as a Major Environmental Variable

The microbiome contributes immensely to an individual’s biotic environment and could be an important environmental variable influencing outcomes to helminth infection. Since these microbial communities are found at epithelial barrier and mucosal surfaces where they can directly interact with helminth parasites, they can shape the tissue micro-environment niches and influence helminth infection outcomes (157–160). For example, the microbiome composition in mice prior to *H. polygyrus* infection or *S. mansoni* infection can influence the worm burden following helminth infection (157, 158). Similarly, oral colonization of mice with commensals such as *Lactobacillus casei*, *Bifidobacterium animalis* prior to helminth infection can alter the outcome of *T. muris* and *Strongyloides venezuelensis* infection in mice (161, 162). Furthermore, treatment of mice with *L. casei*, significantly increase the cecal worm burdens during *T. muris* infection while feeding mice with *B. animalis* significantly reduced *S. venezuelensis* worm burden and egg output (161, 162). There could be direct or indirect effects of these interactions. The effects of the microbiome composition on immune cell populations and epithelial cell function could influence infection outcomes (157, 163, 164). For example, the expression of *Pla2g1b*, an epithelial derived phospholipase A_2_, that is also a host-derived anthelminthic factor is dependent on the intestinal microbiota (163). Similarly, the intestinal microbiome composition and abundance is associated with IL-10 signaling in the host (164). Sometimes, it is mechanistically unclear how microbiome changes can influence susceptibility and pathogenesis of disease during helminth infection, although studies have shown that helminth infection can influence the microbiome composition and diversity in the host recently reviewed here (159). A recent study by Moyat et al., 2022 using germ-free, antibiotic-treated, and specific pathogen-free mice clearly demonstrated that the intestinal bacteria composition can have an impact on host resistance to intestinal helminth *H. polygyrus* (165). Depletion of a complex microbiota through long term-treatment with antibiotics or in germ free mice resulted in more susceptibility to worm infection *via* a mechanism that is dependent on intestinal acetylcholine, a neurotransmitter, necessary for intestinal motility (165). This suggests that the microbiome as a biotic factor, can influence the production of critical signaling molecules necessary for parasite clearance through the “weep and sweep” response. In contrast, *S. mansoni* infection requires a complex microbiome for greater parasite fecundity and pathology during infection (166, 167). Germ-free mice and antibiotic treated mice infected with *S. mansoni* infection resulted in decreased fecal egg counts as well as reduced intestinal pathology and inflammation (166, 167). In summary, the microbiome is a major biotic environmental factor that contributes to inter-individual variation during helminth infection through its effects on the immune and epithelial cell function of the host.

Other studies have suggested that the intestinal bacteria can directly influence parasite establishment, hatchability, and development (168–172). Various *in vitro* and *in vivo* methods demonstrated that the presence of *Escherichia coli*, a common intestinal commensal (173), is important for hatchability of *T. muris* eggs. The role of *E. coli* in parasite establishment is driven by the presence of Type 1 Fimbriae as well as release of microbial byproducts (168, 169). Differences in egg hatching following infection could affect the establishment of the parasite and subsequently the parasite burden, hence the presence or absence of specific commensal bacteria may directly influence parasite burden independently of the host immune response. Experiments with germ free mice or gnotobiotic animals in helminth infection models also demonstrate that the success of parasite infection and fitness is dependent on presence of commensal bacteria (174–178). Therefore, differences in abundance and composition of intestinal bacteria, which could be influenced by use of antibiotics, could contribute to inter-individual variation in parasite burden observed in population studies during helminth infection.

Additionally, the contribution and role of many other components of the microbiome such as fungi, viruses, and archaea (179) in host immune response and parasite development remains poorly understood and whether these components also influence susceptibility and disease pathogenesis during helminth infection needs to be further explored.

## Interplay Between Environment and Host Genetics

While host genetics are important contributors towards response to helminth infections (102). Twin studies suggest that there are both heritable and non-heritable explanations for variation in immune responses (148). However, these two critical factors rarely exist in isolation (142, 180). There are limited studies that assess the additive and interactive effect of environmental and genetic effect on immune responses. As individuals with similar genetic profiles would usually live within the same communities and environment, it is challenging to dissociate one from the other in human studies.

Therefore, mouse models could be helpful in dissociating the complex interactions that exist between genetic and environmental contributors to variation in helminth infection (**Figure 1**). For example, there are indicators that genetic differences in parasite resistance in the laboratory setting may be lost in a natural environment. While *H. polygyrus* infection of BALB/c and C57BL/6 mice in a laboratory setting shows clear differences in resistance to infection, natural infection results in no observable significant differences between these two inbred resistant and susceptible mice (181). Although mechanistically unexplained, a subsequent study suggests that the chronicity of the infection model could explain why the difference was lost between these two different inbred strains of mice (182). In our own studies, differences in susceptibility between wild-type mice and susceptible STAT6^-/-^ knockout mice to *T. muris* infection in the lab setting are no longer observed when these mice are were placed in the re-wilded environment and infected with *T. muris* (147).

Such interactions between genetics and the environment could be complex and mechanistically distinct. As described above, genetic factors such as MHC haplotype may indirectly influence microbial communities within the host (biotic environmental factors) to alter susceptibility to helminth infection (79). How the environmental pressures influences the heritability of helminth resistance genes in the population is also of interest, as shown in a study in sheep whereby significant genotype by environment interactions persists following infection with another helminth parasites (183).

Other host variables such as age, sex, nutritional status could interact with genetics and environmental factors to influence immunity and affect the outcome of helminth infection. A combination of controlled re-wilding experiments (142–144, 147) and perhaps twin studies in helminth endemic populations with detailed questionnaires may provide further insights into the dynamics of interactions of such complex factors.

## Discussions and Conclusions

Environmental exposure and host genetic background are important drivers of inter-individual variation in susceptibility and outcome of helminth parasite infections. Both play a role in driving heterogeneity of responses either as independent variables or through specific interactions that remain poorly understood. Since disease morbidity and parasite burden is observed primarily in small subsets of infected individuals, it is important that necessary resources and research efforts are allocated into studies deciphering how genetic and environmental interactions influences susceptibility to the world’s most neglected disease in human and veterinary medicine. This will require bringing together skillsets and technologies from diverse fields including ecology, quantitative genetics, genomics, immunology, biostatistics, and parasitology. Together, such studies can improve our understanding of key translational factors that regulate immune responses during parasitic helminth infection. Findings from a diverse range of inbred and outbred strains of mice in different environments might provide a more accurate reflection of factors important in diverse human and animal population under free living conditions. There are also opportunities for the identification of new pathways and alleles or regulatory elements that regulate responses in other Type 2 immune mediated diseases such as allergic, metabolic and fibrotic diseases (3, 88). Additionally, there is a need to address the rising incidence of drug resistance seen in the use of anti-helminthics in human and veterinary medicine (184, 185). To further complete this picture, exciting new studies are beginning to show the importance of heterogeneity in helminth parasite genetic factors in the susceptibility and resistance patterns during helminth infection (105–107). The role of parasite genetic diversity in the pathogenesis and outcome of helminth infection, remains a relatively understudied and interesting area to investigate. Parasite genetic heterogeneity could influence the excretory/secretory products produced from these parasites and influence the immunomodulatory properties of these factors. How this might contribute to inter-individual variation to helminth infection is an interesting area to explore.

## Author Disclaimer

The content of this manuscript is solely the responsibility of the authors and does not necessarily represent the views of the National Institute of Health.

## Authors Contributions

OO wrote the first draft of the manuscript. CS and P’NL made significant direct and intellectual contribution to the manuscript. CS made the figure. P’NL edited the manuscript. All authors contributed to the article, read, and approved the submitted version.

## Funding

This work was supported by the Division of Intramural Research, National Institute of Allergy and Infectious Diseases, NIH Order #NLR2110677 and funded the open access publication fees.

## Conflict of Interest

The authors declare that the research was conducted in the absence of any commercial or financial relationships that could be construed as a potential conflict of interest.

The handling editor declared a shared affiliation with the authors at time of review.

## Publisher’s Note

All claims expressed in this article are solely those of the authors and do not necessarily represent those of their affiliated organizations, or those of the publisher, the editors and the reviewers. Any product that may be evaluated in this article, or claim that may be made by its manufacturer, is not guaranteed or endorsed by the publisher.

## References

[1] Quinnell RJ. Genetics of Susceptibility to Human Helminth Infection. Int J Parasitol (2003) 33(11):1219–31. doi: 10.1016/S0020-7519(03)00175-9 13678637

[2] Hotez PJ Brindley PJ Bethony JM King CH Pearce EJ Jacobson J. Helminth Infections: The Great Neglected Tropical Diseases. J Clin Invest (2008) 118(4):1311–21. doi: 10.1172/JCI34261 PMC2276811 18382743

[3] Gause WC Rothlin C Loke PN. Heterogeneity in the Initiation, Development and Function of Type 2 Immunity. Nat Rev Immunol (2020) 20(10):603–14. doi: 10.1038/s41577-020-0301-x PMC9773851 32367051

[4] Girgis NM Gundra UM Loke PN. Immune Regulation During Helminth Infections. PloS Pathogens (2013) 9(4):e1003250. doi: 10.1371/journal.ppat.1003250 23637593 PMC3630086

[5] Douglas B Oyesola O Cooper MM Posey A Wojno ET Giacomin PR. Immune System Investigation Using Parasitic Helminths. Annu Rev Immunol (2021) 39(1):639–65. doi: 10.1146/annurev-immunol-093019-122827 PMC8162934 33646858

[6] Inclan-Rico JM Siracusa MC. First Responders: Innate Immunity to Helminths. Trends Parasitol (2018) 34(10):861–80. doi: 10.1016/j.pt.2018.08.007 PMC6168350 30177466

[7] Allen JE Maizels RM. Diversity and Dialogue in Immunity to Helminths. Nat Rev Immunol (2011) 11(6):375–88. doi: 10.1038/nri2992 21610741

[8] Gause WC Wynn TA Allen JE. Type 2 Immunity and Wound Healing: Evolutionary Refinement of Adaptive Immunity by Helminths. Nat Rev Immunol (2013) 13(8):607–14. doi: 10.1038/nri3476 PMC3789590 23827958

[9] Ajendra J. Lessons in Type 2 Immunity: Neutrophils in Helminth Infections. Semin Immunol (2021) 53:101531. doi: 10.1016/j.smim.2021.101531 34836773

[10] Webb LM Tait Wojno ED. The Role of Rare Innate Immune Cells in Type 2 Immune Activation Against Parasitic Helminths. Parasitology (2017) 144(10):1288–301. doi: 10.1017/S0031182017000488 PMC5962964 28583216

[11] Harris NL Loke P. Recent Advances in Type-2-Cell-Mediated Immunity: Insights From Helminth Infection. Immunity (2017) 47(6):1024–36. doi: 10.1016/j.immuni.2017.11.015 29262347

[12] Oyesola OO Früh SP Webb LM Tait Wojno ED. Cytokines and Beyond: Regulation of Innate Immune Responses During Helminth Infection. Cytokine (2020) 133:154527. doi: 10.1016/j.cyto.2018.08.021 30241895 PMC6422760

[13] McSorley HJ Maizels RM. Helminth Infections and Host Immune Regulation. Clin Microbiol Rev (2012) 25(4):585–608. doi: 10.1128/CMR.05040-11 23034321 PMC3485755

[14] Wynn TA. Type 2 Cytokines: Mechanisms and Therapeutic Strategies. Nat Rev Immunol (2015) 15(5):271–82. doi: 10.1038/nri3831 25882242

[15] Weatherhead JE Gazzinelli-Guimaraes P Knight JM Fujiwara R Hotez PJ Bottazzi ME. Host Immunity and Inflammation to Pulmonary Helminth Infections. Front Immunol (2020) 11:594520–. doi: 10.3389/fimmu.2020.594520 PMC7606285 33193446

[16] Coakley G Harris NL. The Intestinal Epithelium at the Forefront of Host–Helminth Interactions. Trends Parasitol (2020) 36(9):761–72. doi: 10.1016/j.pt.2020.07.002 32713764

[17] Sellers RS Clifford CB Treuting PM Brayton C. Immunological Variation Between Inbred Laboratory Mouse Strains:Points to Consider in Phenotyping Genetically Immunomodified Mice. Veterinary Pathol (2012) 49(1):32–43. doi: 10.1177/0300985811429314 22135019

[18] Hoffmann W Petit G Schulz-Key H Taylor D Bain O Le Goff L. Litomosoides Sigmodontis in Mice: Reappraisal of an Old Model for Filarial Research. Parasitol Today (2000) 16(9):387–9. doi: 10.1016/S0169-4758(00)01738-5 10951598

[19] Graham AL Taylor MD Le Goff L Lamb TJ Magennis M Allen JE. Quantitative Appraisal of Murine Filariasis Confirms Host Strain Differences But Reveals That BALB/c Females are More Susceptible Than Males to Litomosoides Sigmodontis. Microbes Infect (2005) 7(4):612–8. doi: 10.1016/j.micinf.2004.12.019 15820154

[20] Finlay CM Allen JE. The Immune Response of Inbred Laboratory Mice to Litomosoides Sigmodontis: A Route to Discovery in Myeloid Cell Biology. Parasite Immunol (2020) 42(7):e12708. doi: 10.1111/pim.12708 32145033 PMC7317388

[21] Else K Wakelin D. The Effects of H-2 and non-H-2 Genes on the Expulsion of the Nematode Trichuris Muris From Inbred and Congenic Mice. Parasitology (1988) 96(3):543–50. doi: 10.1017/S0031182000080173 3136419

[22] Else K Wakelin D. Genetic Variation in the Humoral Immune Responses of Mice to the Nematode Trichuris Muris. Parasite Immunol (1989) 11(1):77–90. doi: 10.1111/j.1365-3024.1989.tb00650.x 2927957

[23] Else KJ Hültner L Grencis RK. Modulation of Cytokine Production and Response Phenotypes in Murine Trichuriasis. Parasite Immunol (1992) 14(4):441–9. doi: 10.1111/j.1365-3024.1992.tb00018.x 1437236

[24] Behnke JM Wahid FN. Immunological Relationships During Primary Infection With Heligmosomoides Polygyrus (Nematospiroides Dubius): H-2 Linked Genes Determine Worm Survival. Parasitology (1991) 103(1):157–64. doi: 10.1017/S0031182000059400 1945521

[25] Reynolds LA Filbey KJ Maizels RM. Immunity to the Model Intestinal Helminth Parasite Heligmosomoides Polygyrus. Semin Immunopathol (2012) 34(6):829–46. doi: 10.1007/s00281-012-0347-3 PMC3496515 23053394

[26] Filbey KJ Grainger JR Smith KA Boon L van Rooijen N Harcus Y. Innate and Adaptive Type 2 Immune Cell Responses in Genetically Controlled Resistance to Intestinal Helminth Infection. Immunol Cell Biol (2014) 92(5):436–48. doi: 10.1038/icb.2013.109 PMC4038150 24492801

[27] Le Goff L Lamb TJ Graham AL Harcus Y Allen JE. IL-4 is Required to Prevent Filarial Nematode Development in Resistant But Not Susceptible Strains of Mice. Int J Parasitol (2002) 32(10):1277–84. doi: 10.1016/S0020-7519(02)00125-X 12204227

[28] Else KJ Wakelin D Wassom DL Hauda KM. The Influence of Genes Mapping Within the Major Histocompatibility Complex on Resistance to Trichuris Muris Infections in Mice. Parasitology (1990) 101(1):61–7. doi: 10.1017/S0031182000079762 2235076

[29] Else KJ Finkelman FD Maliszewski CR Grencis RK. Cytokine-Mediated Regulation of Chronic Intestinal Helminth Infection. J Exp Med (1994) 179(1):347–51. doi: 10.1084/jem.179.1.347 PMC2191309 8270879

[30] Bancroft AJ Else KJ Sypek JP Grencis RK. Interleukin-12 Promotes a Chronic Intestinal Nematode Infection. Eur J Immunol (1997) 27(4):866–70. doi: 10.1002/eji.1830270410 9130637

[31] Hurst RJM Else KJ. Trichuris Muris Research Revisited: A Journey Through Time. Parasitology (2013) 140(11):1325–39. doi: 10.1017/S0031182013001054 PMC3761323 23965819

[32] Behnke JM Lowe A Clifford S Wakelin D. Cellular and Serological Responses in Resistant and Susceptible Mice Exposed to Repeated Infection With Heligmosomoides Polygyrus Bakeri. Parasite Immunol (2003) 25(6):333–40. doi: 10.1046/j.1365-3024.2003.00639.x 14507331

[33] Behnke JM Mugambi JM Clifford S Iraqi FA Baker RL Gibson JP. Genetic Variation in Resistance to Repeated Infections With Heligmosomoides Polygyrus Bakeri, in Inbred Mouse Strains Selected for the Mouse Genome Project. Parasite Immunol (2006) 28(3):85–94. doi: 10.1111/j.1365-3024.2005.00810.x 16441506

[34] Zhong S Dobson C. Heligmosomoides Polygyrus:Resistance in Inbred, Outbred, and Selected Mice. Exp Parasitol (1996) 82(2):122–31. doi: 10.1006/expr.1996.0016 8617338

[35] Wahid FN Behnke JM Grencis RK Else KJ Ben-Smith AW. Immunological Relationships During Primary Infection With Heligmosomoides Polygyrus: Th2 Cytokines and Primary Response Phenotype. Parasitology (1994) 108(4):461–71. doi: 10.1017/S0031182000076022 8008460

[36] Brailsford TJ Behnke JM. The Dynamics of Trickle Infections With Heligmosomoides Polygyrus in Syngeneic Strains of Mice. Int J Parasitol (1992) 22(3):351–9. doi: 10.1016/S0020-7519(05)80013-X 1639571

[37] Prowse SJ Mitchell GF Ey PL Jenkin CR. The Development of Resistance in Different Inbred Strains of Mice to Infection With Nematospiroides Dubius. Parasite Immunol (1979) 1(4):277–88. doi: 10.1111/j.1365-3024.1979.tb00713.x 551381

[38] Lewis R Behnke JM Cassidy JP Stafford P Murray N Holland CV. The Migration of Ascaris Suum Larvae, and the Associated Pulmonary Inflammatory Response in Susceptible C57BL/6j and Resistant CBA/Ca Mice. Parasitology (2007) 134(9):1301–14. doi: 10.1017/S0031182007002582 17381887

[39] Dold C Cassidy JP Stafford P Behnke JM Holland CV. Genetic Influence on the Kinetics and Associated Pathology of the Early Stage (Intestinal-Hepatic) Migration of Ascaris Suum in Mice. Parasitology (2010) 137(1):173–85. doi: 10.1017/S0031182009990850 19765333

[40] de Oliveira LM Silva Nogueira D Geraldi RM Barbosa FS Amorim CCO Gazzinelli-Guimarães AC. Genetic Background Affects the Mucosal SIgA Levels, Parasite Burden, Lung Inflammation and Susceptibility of Male Mice to Ascaris Suum Infection. Infect Immun (2021) 90(2):Iai0059521. doi: 10.1128/IAI.00595-21 PMC8852741 34807734

[41] Knott ML Hogan SP Wang H Matthaei KI Dent LA. FVB/N Mice are Highly Resistant to Primary Infection With Nippostrongylus Brasiliensis. Parasitology (2009) 136(1):93–106. doi: 10.1017/S0031182008005192 19126273 PMC2728364

[42] Stadnyk AW McElroy PJ Gauldie J Befus AD. Characterization of Nippostrongylus Brasiliensis Infection in Different Strains of Mice. J Parasitol (1990) 76(3):377–82. doi: 10.2307/3282670 2352068

[43] Harvie M Camberis M Tang S-C Delahunt B Paul W Gros GL. The Lung Is an Important Site for Priming CD4 T-Cell-Mediated Protective Immunity Against Gastrointestinal Helminth Parasites. Infect Immun (2010) 78(9):3753–62. doi: 10.1128/IAI.00502-09 PMC2937440 20605978

[44] Sciutto E Fragoso G Diaz ML Valdez F Montoya RM Govezensky T. MurineTaenia Crassiceps Cysticercosis: H-2 Complex and Sex Influence on Susceptibility. Parasitol Res (1991) 77(3):243–6. doi: 10.1007/BF00930866 2047372

[45] Fragoso G Lamoyi E Mellor A Lomeli C Govezensky T Sciutto E. Genetic Control of Susceptibility to Taenia Crassiceps Cysticercosis. Parasitology (1996) 112(1):119–24. doi: 10.1017/S003118200006515X 8587794

[46] López-Briones S Soloski M Bojalil R Fragoso G Sciutto E. CD4+ Tcrαβ T Cells are Critically Involved in the Control of Experimental Murine Cysticercosis in C57BL/6J Mice. Parasitol Res (2001) 87(10):826–32. doi: 10.1007/s004360100448 11688888

[47] López-Briones S Lamoyi E Fragoso G Soloski MJ Sciutto E. Taenia Crassiceps Cysticercosis: Immune Response in Susceptible and Resistant BALB/c Mouse Substrains. Parasitol Res (2003) 90(3):236–42. doi: 10.1007/s00436-003-0848-z 12783314

[48] Adalid-Peralta L Lopez-Roblero A Camacho-Vázquez C Nájera-Ocampo M Guevara-Salinas A Ruiz-Monroy N. Regulatory T Cells as an Escape Mechanism to the Immune Response in Taenia Crassiceps Infection. Front Cell Infect Microbiol (2021) 11:630583–. doi: 10.3389/fcimb.2021.630583 PMC8076859 33928043

[49] Wakelin D. Genetic Variation in Resistance to Parasitic Infection: Experimental Approaches and Practical Applications. Res Veterinary Sci (1992) 53(2):139–47. doi: 10.1016/0034-5288(92)90101-7 1439201

[50] Shainheit MG Lasocki KW Finger E Larkin BM Smith PM Sharpe AH. The Pathogenic Th17 Cell Response to Major Schistosome Egg Antigen is Sequentially Dependent on IL-23 and IL-1β. J Immunol (2011) 187(10):5328–35. doi: 10.4049/jimmunol.1101445 PMC3653625 22003203

[51] Ponichtera HE Shainheit MG Liu BC Raychowdhury R Larkin BM Russo JM. CD209a Expression on Dendritic Cells Is Critical for the Development of Pathogenic Th17 Cell Responses in Murine Schistosomiasis. J Immunol (2014) 192(10):4655–65. doi: 10.4049/jimmunol.1400121 PMC4030302 24729611

[52] Kalantari P Morales Y Miller EA Jaramillo LD Ponichtera HE Wuethrich MA. CD209a Synergizes With Dectin-2 and Mincle to Drive Severe Th17 Cell-Mediated Schistosome Egg-Induced Immunopathology. Cell Rep (2018) 22(5):1288–300. doi: 10.1016/j.celrep.2018.01.001 PMC5815841 29386115

[53] Smith PM Sproule TJ Philip VM Roopenian DC Stadecker MJ. Minor Genomic Differences Between Related B6 and B10 Mice Affect Severity of Schistosome Infection by Governing the Mode of Dendritic Cell Activation. Eur J Immunol (2015) 45(8):2312–23. doi: 10.1002/eji.201545547 PMC4597890 25959828

[54] Mbow M Larkin BM Meurs L Wammes LJ de Jong SE Labuda LA. T-Helper 17 Cells are Associated With Pathology in Human Schistosomiasis. J Infect Dis (2013) 207(1):186–95. doi: 10.1093/infdis/jis654 PMC3571236 23087431

[55] von Moltke J Pepper M. Sentinels of the Type 2 Immune Response. Trends Immunol (2018) 39(2):99–111. doi: 10.1016/j.it.2017.10.004 29122456 PMC6181126

[56] El-Naccache DW Haskó G Gause WC. Early Events Triggering the Initiation of a Type 2 Immune Response. Trends Immunol (2021) 42(2):151–64. doi: 10.1016/j.it.2020.11.006 PMC9813923 33386241

[57] Faniyi AA Wijanarko KJ Tollitt J Worthington JJ. Helminth Sensing at the Intestinal Epithelial Barrier—A Taste of Things to Come. Front Immunol (2020) 11(1489). doi: 10.3389/fimmu.2020.01489 PMC7409516 32849506

[58] Howitt MR Cao YG Gologorsky MB Li JA Haber AL Biton M. The Taste Receptor TAS1R3 Regulates Small Intestinal Tuft Cell Homeostasis. ImmunoHorizons (2020) 4(1):23–32. doi: 10.4049/immunohorizons.1900099 31980480 PMC7197368

[59] Rajeev S Sosnowski O Li S Allain T Buret AG McKay DM. Enteric Tuft Cells in Host-Parasite Interactions. Pathogens (2021) 10(9):1163. doi: 10.3390/pathogens10091163 34578195 PMC8467374

[60] Stear MJ Boag B Cattadori I Murphy L. Genetic Variation in Resistance to Mixed, Predominantly Teladorsagia Circumcincta Nematode Infections of Sheep: From Heritabilities to Gene Identification. Parasite Immunol (2009) 31(5):274–82. doi: 10.1111/j.1365-3024.2009.01105.x 19388948

[61] Sayers G Good B Hanrahan JP Ryan M Sweeney T. Intron 1 of the Interferon Gamma Gene: Its Role in Nematode Resistance in Suffolk and Texel Sheep Breeds. Res Vet Sci (2005) 79(3):191–6. doi: 10.1016/j.rvsc.2004.12.002 16054889

[62] Coltman DW Wilson K Pilkington JG Stear MJ Pemberton JM. A Microsatellite Polymorphism in the Gamma Interferon Gene Is Associated With Resistance to Gastrointestinal Nematodes in a Naturally-Parasitized Population of Soay Sheep. Parasitology (2001) 122(Pt 5):571–82. doi: 10.1017/S0031182001007570 11393831

[63] Ezenwa VO Etienne RS Luikart G Beja-Pereira A Jolles A. Hidden Consequences of Living in a Wormy World: Nematode-Induced Immune Suppression Facilitates Tuberculosis Invasion in African Buffalo. Am Nat (2010) 176(5):613–24. doi: 10.1086/656496 20849271

[64] Ezenwa VO Budischak SA Buss P Seguel M Luikart G Jolles AE. Natural Resistance to Worms Exacerbates Bovine Tuberculosis Severity Independently of Worm Coinfection. Proc Natl Acad Sci USA (2021) 118(3):e2015080118. doi: 10.1073/pnas.2015080118 33431676 PMC7826365

[65] Mcneilly TN Devaney E Matthews JB. Teladorsagia Circumcincta in the Sheep Abomasum: Defining the Role of Dendritic Cells in T Cell Regulation and Protective Immunity. Parasite Immunol (2009) 31(7):347–56. doi: 10.1111/j.1365-3024.2009.01110.x 19527450

[66] Williams-Blangero S Subedi J Upadhayay RP Manral DB Rai DR Jha B. Genetic Analysis of Susceptibility to Infection With Ascaris Lumbricoides. Am J Trop Med Hygiene (1999) 60(6):921–6. doi: 10.4269/ajtmh.1999.60.921 10403321

[67] Williams-Blangero S VandeBerg JL Subedi J Aivaliotis MJ Rai DR Upadhayay RP. Genes on Chromosomes 1 and 13 Have Significant Effects on Ascaris Infection. Proc Natl Acad Sci (2002) 99(8):5533–8. doi: 10.1073/pnas.082115999 PMC122804 11960011

[68] Williams-Blangero S VandeBerg JL Subedi J Jha B Corrêa-Oliveira R Blangero J. Localization of Multiple Quantitative Trait Loci Influencing Susceptibility to Infection With Ascaris Lumbricoides. J Infect Dis (2008) 197(1):66–71. doi: 10.1086/524060 18171287

[69] Holland CV. Predisposition to Ascariasis: Patterns, Mechanisms and Implications. Parasitology (2009) 136(12):1537–47. doi: 10.1017/S0031182009005952 19450374

[70] Yang M Sun L Wang S Ko K-H Xu H Zheng B-J. Cutting Edge: Novel Function of B Cell-Activating Factor in the Induction of IL-10–Producing Regulatory B Cells. J Immunol (2010) 184(7):3321–5. doi: 10.4049/jimmunol.0902551 20208006

[71] Hussaarts L van der Vlugt LEPM Yazdanbakhsh M Smits HH. Regulatory B-Cell Induction by Helminths: Implications for Allergic Disease. J Allergy Clin Immunol (2011) 128(4):733–9. doi: 10.1016/j.jaci.2011.05.012 21684587

[72] Williams-Blangero S McGarvey ST Subedi J Wiest PM Upadhayay RP Rai DR. Genetic Component to Susceptibility to Trichuris Trichiura: Evidence From Two Asian Populations. Genet Epidemiol: Off Publ Int Genet Epidemiol Soc (2002) 22(3):254–64. doi: 10.1002/gepi.0187 11921085

[73] Williams-Blangero S VandeBerg JL Subedi J Jha B Dyer TD Blangero J. Two Quantitative Trait Loci Influence Whipworm (Trichuris Trichiura) Infection in a Nepalese Population. J Infect Dis (2008) 197(8):1198–203. doi: 10.1086/533493 PMC4122289 18462166

[74] Marquet S Abel L Hillaire D Dessein H Kalil J Feingold J. Genetic Localization of a Locus Controlling the Intensity of Infection by Schistosoma Mansoni on Chromosome 5q31–Q33. Nat Genet (1996) 14(2):181–4. doi: 10.1038/ng1096-181 8841190

[75] Zinn-Justin A Marquet S Hillaire D Dessein A Abel L. Genome Search for Additional Human Loci Controlling Infection Levels by Schistosoma Mansoni. Am J Trop Med Hygiene (2001) 65(6):754–8. doi: 10.4269/ajtmh.2001.65.754 11791970

[76] Quinnell RJ Pullan RL Breitling LP Geiger SM Cundill B Correa-Oliveira R. Genetic and Household Determinants of Predisposition to Human Hookworm Infection in a Brazilian Community. J Infect Dis (2010) 202(6):954–61. doi: 10.1086/655813 PMC2938471 20681887

[77] Mewamba EM Nyangiri OA Noyes HA Egesa M Matovu E Simo G. The Genetics of Human Schistosomiasis Infection Intensity and Liver Disease: A Review. Front Immunol (2021) 12. doi: 10.3389/fimmu.2021.613468 PMC7917240 33659002

[78] Dessein H Duflot N Romano A Opio C Pereira V Mola C. Genetic Algorithms Identify Individuals With High Risk of Severe Liver Disease Caused by Schistosomes. Hum Genet (2020) 139(6):821–31. doi: 10.1007/s00439-020-02160-4 32277285

[79] Montero BK Wasimuddin Schwensow N Gillingham MAF Ratovonamana YR Rakotondranary SJ. Evidence of MHC Class I and II Influencing Viral and Helminth Infection *via* the Microbiome in a non-Human Primate. PloS Pathogens (2021) 17(11):e1009675. doi: 10.1371/journal.ppat.1009675 34748618 PMC8601626

[80] Kubinak JL Stephens WZ Soto R Petersen C Chiaro T Gogokhia L. MHC Variation Sculpts Individualized Microbial Communities That Control Susceptibility to Enteric Infection. Nat Commun (2015) 6(1):8642. doi: 10.1038/ncomms9642 26494419 PMC4621775

[81] Khan AA Yurkovetskiy L O'Grady K Pickard JM de Pooter R Antonopoulos DA. Polymorphic Immune Mechanisms Regulate Commensal Repertoire. Cell Rep (2019) 29(3):541–50.e4. doi: 10.1016/j.celrep.2019.09.010 31618625 PMC6904226

[82] Bolnick DI Snowberg LK Caporaso JG Lauber C Knight R Stutz WE. Major Histocompatibility Complex Class IIb Polymorphism Influences Gut Microbiota Composition and Diversity. Mol Ecol (2014) 23(19):4831–45. doi: 10.1111/mec.12846 24975397

[83] Motran CC Silvane L Chiapello LS Theumer MG Ambrosio LF Volpini X. Helminth Infections: Recognition and Modulation of the Immune Response by Innate Immune Cells. Front Immunol (2018) 9. doi: 10.3389/fimmu.2018.00664 PMC5893867 29670630

[84] Perrigoue JG Marshall FA Artis D. On the Hunt for Helminths: Innate Immune Cells in the Recognition and Response to Helminth Parasites. Cell Microbiol (2008) 10(9):1757–64. doi: 10.1111/j.1462-5822.2008.01174.x PMC2683372 18505479

[85] Urban JF Noben-Trauth N Donaldson DD Madden KB Morris SC Collins M. IL-13, IL-4rα, and Stat6 Are Required for the Expulsion of the Gastrointestinal Nematode Parasite Nippostrongylus Brasiliensis. Immunity (1998) 8(2):255–64. doi: 10.1016/S1074-7613(00)80477-X 9492006

[86] Finkelman FD Shea-Donohue T Goldhill J Sullivan CA Morris SC Madden KB. CYTOKINE REGULATION OF HOST DEFENSE AGAINST PARASITIC GASTROINTESTINAL NEMATODES:Lessons From Studies With Rodent Models*. Annu Rev Immunol (1997) 15(1):505–33. doi: 10.1146/annurev.immunol.15.1.505 9143698

[87] von Moltke J O’Leary CE Barrett NA Kanaoka Y Austen KF Locksley RM. Leukotrienes Provide an NFAT-Dependent Signal That Synergizes With IL-33 to Activate ILC2s. J Exp Med (2016) 214(1):27–37. doi: 10.1084/jem.20161274 28011865 PMC5206504

[88] Gieseck RL Wilson MS Wynn TA. Type 2 Immunity in Tissue Repair and Fibrosis. Nat Rev Immunol (2018) 18(1):62–76. doi: 10.1038/nri.2017.90 28853443

[89] Sorobetea D Svensson-Frej M Grencis R. Immunity to Gastrointestinal Nematode Infections. Mucosal Immunol (2018) 11(2):304–15. doi: 10.1038/mi.2017.113 29297502

[90] Grencis RK. Immunity to Helminths: Resistance, Regulation, and Susceptibility to Gastrointestinal Nematodes. Annu Rev Immunol (2015) 33:201–25. doi: 10.1146/annurev-immunol-032713-120218 25533702

[91] Alves CC Araujo N Cassali GD Fonseca CT. Parasitological, Pathological, and Immunological Parameters Associated With Schistosoma Mansoni Infection and Reinfection in BALB/c AND C57BL/6 Mice. J Parasitol (2016) 102(3):336–41, 6. doi: 10.1645/14-664 26928866

[92] Dessein AJ Hillaire D Elwali NEMA Marquet S Mohamed-Ali Q Mirghani A. Severe Hepatic Fibrosis in Schistosoma Mansoni Infection Is Controlled by a Major Locus That Is Closely Linked to the Interferon-γ Receptor Gene. Am J Hum Genet (1999) 65(3):709–21. doi: 10.1086/302526 PMC1377977 10441577

[93] Sertorio M Hou X Carmo RF Dessein H Cabantous S Abdelwahed M. IL-22 and IL-22 Binding Protein (IL-22BP) Regulate Fibrosis and Cirrhosis in Hepatitis C Virus and Schistosome Infections. Hepatology (2015) 61(4):1321–31. doi: 10.1002/hep.27629 25476703

[94] Dessein A Chevillard C Arnaud V Hou X Hamdoun AA Dessein H. Variants of CTGF Are Associated With Hepatic Fibrosis in Chinese, Sudanese, and Brazilians Infected With Schistosomes. J Exp Med (2009) 206(11):2321–8. doi: 10.1084/jem.20090383 PMC2768853 19822645

[95] Dessein A Arnaud V He H Li J Dessein H Hou X. Genetic Analysis of Human Predisposition to Hepatosplenic Disease Caused by Schistosomes Reveals the Crucial Role of Connective Tissue Growth Factor in Rapid Progression to Severe Hepatic Fibrosis. Pathol Biol (2013) 61(1):3–10. doi: 10.1016/j.patbio.2013.01.005 23414795

[96] Bancroft AJ Artis D Donaldson DD Sypek JP Grencis RK. Gastrointestinal Nematode Expulsion in IL-4 Knockout Mice is IL-13 Dependent. Eur J Immunol (2000) 30(7):2083–91. doi: 10.1002/1521-4141(200007)30:7<2083::AID-IMMU2083>3.0.CO;2-3 10940898

[97] Mountford AP Hogg KG Coulson PS Brombacher F. Signaling *via* Interleukin-4 Receptor Alpha Chain is Required for Successful Vaccination Against Schistosomiasis in BALB/c Mice. Infect Immun (2001) 69(1):228–36. doi: 10.1128/IAI.69.1.228-236.2001 PMC97876 11119510

[98] Kalantari P Bunnell SC Stadecker MJ. The C-Type Lectin Receptor-Driven, Th17 Cell-Mediated Severe Pathology in Schistosomiasis: Not All Immune Responses to Helminth Parasites Are Th2 Dominated. Front Immunol (2019) 10. doi: 10.3389/fimmu.2019.00026 PMC6363701 30761125

[99] Fumagalli M Pozzoli U Cagliani R Comi GP Riva S Clerici M. Parasites Represent a Major Selective Force for Interleukin Genes and Shape the Genetic Predisposition to Autoimmune Conditions. J Exp Med (2009) 206(6):1395–408. doi: 10.1084/jem.20082779 PMC2715056 19468064

[100] Carneiro VL da Silva HBF Queiroz GA Veiga RV Oliveira PRS Carneiro NVQ. WSB1 and IL21R Genetic Variants Are Involved in Th2 Immune Responses to Ascaris Lumbricoides. Front Immunol (2021) 12:622051. doi: 10.3389/fimmu.2021.622051 33692795 PMC7937724

[101] Figueiredo CA Barreto ML Alcantara-Neves NM Rodrigues LC Cooper PJ Cruz AA. Coassociations Between IL10 Polymorphisms, IL-10 Production, Helminth Infection, and Asthma/Wheeze in an Urban Tropical Population in Brazil. J Allergy Clin Immunol (2013) 131(6):1683–90. doi: 10.1016/j.jaci.2012.10.043 PMC5017514 23273955

[102] Fumagalli M Pozzoli U Cagliani R Comi GP Bresolin N Clerici M. The Landscape of Human Genes Involved in the Immune Response to Parasitic Worms. BMC Evol Biol (2010) 10(1):264. doi: 10.1186/1471-2148-10-264 20807397 PMC2940816

[103] Peisong G Mao XQ Enomoto T Feng Z Gloria-Bottini F Bottini E. An Asthma-Associated Genetic Variant of STAT6 Predicts Low Burden of Ascaris Worm Infestation. Genes Immun (2004) 5(1):58–62. doi: 10.1038/sj.gene.6364030 14735150

[104] Moller M Gravenor MB Roberts SE Sun D Gao P Hopkin JM. Genetic Haplotypes of Th-2 Immune Signalling Link Allergy to Enhanced Protection to Parasitic Worms. Hum Mol Genet (2007) 16(15):1828–36. doi: 10.1093/hmg/ddm131 17519224

[105] Nejsum P Roepstorff A Anderson TJC JØRgensen C Fredholm M Thamsborg SM. The Dynamics of Genetically Marked Ascaris Suum Infections in Pigs. Parasitology (2009) 136(2):193–201. doi: 10.1017/S0031182008005349 19091154

[106] Le Clec'h W Chevalier FD McDew-White M Menon V Arya GA Anderson TJC. Genetic Architecture of Transmission Stage Production and Virulence in Schistosome Parasites. Virulence (2021) 12(1):1508–26. doi: 10.1080/21505594.2021.1932183 PMC8237990 34167443

[107] Bellaby T Robinson K Wakelin D. Induction of Differential T-Helper-Cell Responses in Mice Infected With Variants of the Parasitic Nematode Trichuris Muris. Infect Immun (1996) 64(3):791–5. doi: 10.1128/iai.64.3.791-795.1996 PMC173839 8641783

[108] Bourke CD Maizels RM Mutapi F. Acquired Immune Heterogeneity and its Sources in Human Helminth Infection. Parasitology (2011) 138(2):139–59. doi: 10.1017/S0031182010001216 PMC3021922 20946693

[109] Zarowiecki M Berriman M. What Helminth Genomes Have Taught Us About Parasite Evolution. Parasitology (2015) 142 Suppl 1(Suppl 1):S85–97. doi: 10.1017/S0031182014001449 PMC4413821 25482650

[110] Hewitson JP Grainger JR Maizels RM. Helminth Immunoregulation: The Role of Parasite Secreted Proteins in Modulating Host Immunity. Mol Biochem Parasitol (2009) 167(1):1–11. doi: 10.1016/j.molbiopara.2009.04.008 19406170 PMC2706953

[111] Mutapi F Ndhlovu P Hagan P Woolhouse M. A Comparison of Humoral Responses to Schistosoma Haematobium in Areas With Low and High Levels of Infection. Parasite Immunol (1997) 19(6):255–63. doi: 10.1046/j.1365-3024.1997.d01-206.x 9364555

[112] Acosta LP McManus DP Aligui GD Olveda RM Tiu WU. Antigen-Specific Antibody Isotype Patterns to Schistosoma Japonicum Recombinant and Native Antigens in a Defined Population in Leyte, the Philippines. Am J Trop Med Hygiene (2004) 70(5):549–55. doi: 10.4269/ajtmh.2004.70.549 15155990

[113] Johnston CE Bradley JE Behnke JM Matthews KR Else KJ. Isolates of Trichuris Muris Elicit Different Adaptive Immune Responses in Their Murine Host. Parasite Immunol (2005) 27(3):69–78. doi: 10.1111/j.1365-3024.2005.00746.x 15882233

[114] Koyama K Ito Y. Comparative Studies on Immune Responses to Infection in Susceptible B10.BR Mice Infected With Different Strains of the Murine Nematode Parasite Trichuris Muris. Parasite Immunol (1996) 18(5):257–63. doi: 10.1046/j.1365-3024.1996.d01-92.x 9229378

[115] Pearce EJ MacDonald AS. The Immunobiology of Schistosomiasis. Nat Rev Immunol (2002) 2(7):499–511. doi: 10.1038/nri843 12094224

[116] Wynn TA Oswald IP Eltoum IA Caspar P Lowenstein CJ Lewis FA. Elevated Expression of Th1 Cytokines and Nitric Oxide Synthase in the Lungs of Vaccinated Mice After Challenge Infection With Schistosoma Mansoni. J Immunol (1994) 153(11):5200–9. 7525727

[117] Jenkins SJ Hewitson JP Jenkins GR Mountford AP. Modulation of the Host's Immune Response by Schistosome Larvae. Parasite Immunol (2005) 27(10-11):385–93. doi: 10.1111/j.1365-3024.2005.00789.x PMC1825761 16179032

[118] Cuesta-Astroz Y Oliveira FSD Nahum LA Oliveira G. Helminth Secretomes Reflect Different Lifestyles and Parasitized Hosts. Int J Parasitol (2017) 47(9):529–44. doi: 10.1016/j.ijpara.2017.01.007 28336271

[119] Tsai IJ Zarowiecki M Holroyd N Garciarrubio A Sanchez-Flores A Brooks KL. The Genomes of Four Tapeworm Species Reveal Adaptations to Parasitism. Nature (2013) 496(7443):57–63. doi: 10.1038/nature12031 23485966 PMC3964345

[120] Salgame P Yap GS Gause WC. Effect of Helminth-Induced Immunity on Infections With Microbial Pathogens. Nat Immunol (2013) 14(11):1118–26. doi: 10.1038/ni.2736 PMC4955540 24145791

[121] Desai P Diamond MS Thackray LB. Helminth-Virus Interactions: Determinants of Coinfection Outcomes. Gut Microbes (2021) 13(1):1961202–. doi: 10.1080/19490976.2021.1961202 PMC8405156 34428107

[122] Mabbott NA. The Influence of Parasite Infections on Host Immunity to Co-Infection With Other Pathogens. Front Immunol (2018) 9:2579–. doi: 10.3389/fimmu.2018.02579 PMC6237250 30467504

[123] Anthony RM Urban JF Alem F Hamed HA Rozo CT Boucher J-L. Memory TH2 Cells Induce Alternatively Activated Macrophages to Mediate Protection Against Nematode Parasites. Nat Med (2006) 12(8):955–60. doi: 10.1038/nm1451 PMC1955764 16892038

[124] Chen F Wu W Millman A Craft JF Chen E Patel N. Neutrophils Prime a Long-Lived Effector Macrophage Phenotype That Mediates Accelerated Helminth Expulsion. Nat Immunol (2014) 15(10):938–46. doi: 10.1038/ni.2984 PMC4479254 25173346

[125] Obata-Ninomiya K Ishiwata K Tsutsui H Nei Y Yoshikawa S Kawano Y. The Skin is an Important Bulwark of Acquired Immunity Against Intestinal Helminths. J Exp Med (2013) 210(12):2583–95. doi: 10.1084/jem.20130761 PMC3832932 24166714

[126] Bickle QD Solum J Helmby H. Chronic Intestinal Nematode Infection Exacerbates Experimental Schistosoma Mansoni Infection. Infect Immun (2008) 76(12):5802–9. doi: 10.1128/IAI.00827-08 PMC2583585 18824532

[127] Yap GS Gause WC. Helminth Infections Induce Tissue Tolerance Mitigating Immunopathology But Enhancing Microbial Pathogen Susceptibility. Front Immunol (2018) 9:2135–. doi: 10.3389/fimmu.2018.02135 PMC6198046 30386324

[128] Rajamanickam A Munisankar S Dolla C Menon PA Nutman TB Babu S. Helminth Coinfection Alters Monocyte Activation, Polarization, and Function in Latent Mycobacterium Tuberculosis Infection. J Immunol (2020) 204(5):1274–86. doi: 10.4049/jimmunol.1901127 PMC7033029 31953351

[129] Osborne LC Monticelli LA Nice TJ Sutherland TE Siracusa MC Hepworth MR. Virus-Helminth Coinfection Reveals a Microbiota-Independent Mechanism of Immunomodulation. Science (2014) 345(6196):578–82. doi: 10.1126/science.1256942 PMC4548887 25082704

[130] Reese TA Wakeman BS Choi HS Hufford MM Huang SC Zhang X. Helminth Infection Reactivates Latent γ-Herpesvirus *via* Cytokine Competition at a Viral Promoter. Science (2014) 345(6196):573–7. doi: 10.1126/science.1254517 PMC4531374 24968940

[131] Desai P Janova H White JP Reynoso GV Hickman HD Baldridge MT. Enteric Helminth Coinfection Enhances Host Susceptibility to Neurotropic Flaviviruses *via* a Tuft Cell-IL-4 Receptor Signaling Axis. Cell (2021) 184(5):1214–31.e16. doi: 10.1016/j.cell.2021.01.051 33636133 PMC7962748

[132] Reynolds LA Redpath SA Yurist-Doutsch S Gill N Brown EM van der Heijden J. Enteric Helminths Promote Salmonella Coinfection by Altering the Intestinal Metabolome. J Infect Dis (2017) 215(8):1245–54. doi: 10.1093/infdis/jix141 PMC5853568 28368463

[133] Blackwell AD Martin M Kaplan H Gurven M. Antagonism Between Two Intestinal Parasites in Humans: The Importance of Co-Infection for Infection Risk and Recovery Dynamics. Proc Biol Sci (2013) 280(1769):20131671–. doi: 10.1098/rspb.2013.1671 PMC3768312 23986108

[134] Diallo TO Remoue F Schacht AM Charrier N Dompnier JP Pillet S. Schistosomiasis Co-Infection in Humans Influences Inflammatory Markers in Uncomplicated Plasmodium Falciparum Malaria. Parasite Immunol (2004) 26(8-9):365–9. doi: 10.1111/j.0141-9838.2004.00719.x 15679634

[135] Gause WC Maizels RM. Macrobiota - Helminths as Active Participants and Partners of the Microbiota in Host Intestinal Homeostasis. Curr Opin Microbiol (2016) 32:14–8. doi: 10.1016/j.mib.2016.04.004 PMC4983462 27116368

[136] Loke P Lim YAL. Helminths and the Microbiota: Parts of the Hygiene Hypothesis. Parasite Immunol (2015) 37(6):314–23. doi: 10.1111/pim.12193 PMC4428757 25869420

[137] Ahmed N French T Rausch S Kühl A Hemminger K Dunay IR. Toxoplasma Co-Infection Prevents Th2 Differentiation and Leads to a Helminth-Specific Th1 Response. Front Cell Infect Microbiol (2017) 7. doi: 10.3389/fcimb.2017.00341 PMC5524676 28791259

[138] Miller CM Smith NC Ikin RJ Boulter NR Dalton JP Donnelly S. Immunological Interactions Between 2 Common Pathogens, Th1-Inducing Protozoan Toxoplasma Gondii and the Th2-Inducing Helminth Fasciola Hepatica. PloS One (2009) 4(5):e5692. doi: 10.1371/journal.pone.0005692 19478853 PMC2682559

[139] Liesenfeld O Dunay IR Erb KJ. Infection With Toxoplasma Gondii Reduces Established and Developing Th2 Responses Induced by Nippostrongylus Brasiliensis Infection. Infect Immun (2004) 72(7):3812–22. doi: 10.1128/IAI.72.7.3812-3822.2004 PMC427426 15213122

[140] Hoeve MA Mylonas KJ Fairlie-Clarke KJ Mahajan SM Allen JE Graham AL. Plasmodium Chabaudi Limits Early Nippostrongylus Brasiliensis-Induced Pulmonary Immune Activation and Th2 Polarization in Co-Infected Mice. BMC Immunol (2009) 10:60. doi: 10.1186/1471-2172-10-60 19951425 PMC3224723

[141] Montes M Sanchez C Verdonck K Lake JE Gonzalez E Lopez G. Regulatory T Cell Expansion in HTLV-1 and Strongyloidiasis Co-Infection is Associated With Reduced IL-5 Responses to Strongyloides Stercoralis Antigen. PloS Neglected Trop Dis (2009) 3(6):e456–e. doi: 10.1371/journal.pntd.0000456 PMC2686100 19513105

[142] Graham AL. Naturalizing Mouse Models for Immunology. Nat Immunol (2021) 22(2):111–7. doi: 10.1038/s41590-020-00857-2 33495644

[143] Yeung F Chen Y-H Lin J-D Leung JM McCauley C Devlin JC. Altered Immunity of Laboratory Mice in the Natural Environment Is Associated With Fungal Colonization. Cell Host Microbe (2020) 27(5):809–22.e6. doi: 10.1016/j.chom.2020.02.015 32209432 PMC7276265

[144] Lin J-D Devlin JC Yeung F McCauley C Leung JM Chen Y-H. Rewilding Nod2 and Atg16l1 Mutant Mice Uncovers Genetic and Environmental Contributions to Microbial Responses and Immune Cell Composition. Cell Host Microbe (2020) 27(5):830–40.e4. doi: 10.1016/j.chom.2020.03.001 32209431 PMC7228860

[145] Beura LK Hamilton SE Bi K Schenkel JM Odumade OA Casey KA. Normalizing the Environment Recapitulates Adult Human Immune Traits in Laboratory Mice. Nature (2016) 532(7600):512–6. doi: 10.1038/nature17655 PMC4871315 27096360

[146] Rosshart SP Herz J Vassallo BG Hunter A Wall MK Badger JH. Laboratory Mice Born to Wild Mice Have Natural Microbiota and Model Human Immune Responses. Science (2019) 365(6452):eaaw4361. doi: 10.1126/science.aaw4361 31371577 PMC7377314

[147] Leung JM Budischak SA Chung The H Hansen C Bowcutt R Neill R. Rapid Environmental Effects on Gut Nematode Susceptibility in Rewilded Mice. PloS Biol (2018) 16(3):e2004108. doi: 10.1371/journal.pbio.2004108 29518091 PMC5843147

[148] Brodin P Jojic V Gao T Bhattacharya S Angel Cesar JL Furman D. Variation in the Human Immune System Is Largely Driven by Non-Heritable Influences. Cell (2015) 160(1):37–47. doi: 10.1016/j.cell.2014.12.020 25594173 PMC4302727

[149] Bär J Leung JM Hansen C Loke P Hall AR Conour L. Strong Effects of Lab-to-Field Environmental Transitions on the Bacterial Intestinal Microbiota of Mus Musculus are Modulated by Trichuris Murisinfection. FEMS Microbiol Ecol (2020) 96(10). doi: 10.1093/femsec/fiaa167 32816007

[150] Mair I McNeilly TN Corripio-Miyar Y Forman R Else KJ. Embracing Nature's Complexity: Immunoparasitology in the Wild. Semin Immunol (2021) 53:101525. doi: 10.1016/j.smim.2021.101525 34785137 PMC8713030

[151] Rosshart SP Vassallo BG Angeletti D Hutchinson DS Morgan AP Takeda K. Wild Mouse Gut Microbiota Promotes Host Fitness and Improves Disease Resistance. Cell (2017) 171(5):1015–28.e13. doi: 10.1016/j.cell.2017.09.016 29056339 PMC6887100

[152] Hamilton SE Badovinac VP Beura LK Pierson M Jameson SC Masopust D. New Insights Into the Immune System Using Dirty Mice. J Immunol (2020) 205(1):3–11. doi: 10.4049/jimmunol.2000171 32571979 PMC7316151

[153] Fiege JK Block KE Pierson MJ Nanda H Shepherd FK Mickelson CK. Mice With Diverse Microbial Exposure Histories as a Model for Preclinical Vaccine Testing. Cell Host Microbe (2021) 29(12):1815–27.e6. doi: 10.1016/j.chom.2021.10.001 34731647 PMC8665115

[154] Lindner C Thomsen I Wahl B Ugur M Sethi MK Friedrichsen M. Diversification of Memory B Cells Drives the Continuous Adaptation of Secretory Antibodies to Gut Microbiota. Nat Immunol (2015) 16(8):880–8. doi: 10.1038/ni.3213 26147688

[155] Ma J Classon CH Stark JM Li M Huang H-J Vrtala S. Laboratory Mice With a Wild Microbiota Generate Strong Allergic Immune Responses. bioRxiv (2021), 2021.03.28.437143. doi: 10.1101/2021.03.28.437143 37774008

[156] Reese Tiffany A Bi K Kambal A Filali-Mouhim A Beura Lalit K Bürger Matheus C. Sequential Infection With Common Pathogens Promotes Human-Like Immune Gene Expression and Altered Vaccine Response. Cell Host Microbe (2016) 19(5):713–9. doi: 10.1016/j.chom.2016.04.003 PMC4896745 27107939

[157] Reynolds LA Smith KA Filbey KJ Harcus Y Hewitson JP Redpath SA. Commensal-Pathogen Interactions in the Intestinal Tract. Gut Microbes (2014) 5(4):522–32. doi: 10.4161/gmic.32155 PMC4822684 25144609

[158] Cortés A Clare S Costain A Almeida A McCarthy C Harcourt K. Baseline Gut Microbiota Composition Is Associated With Schistosoma Mansoni Infection Burden in Rodent Models. Front Immunol (2020) 11(2988). doi: 10.3389/fimmu.2020.593838 PMC7718013 33329584

[159] Rapin A Harris NL. Helminth–Bacterial Interactions: Cause and Consequence. Trends Immunol (2018) 39(9):724–33. doi: 10.1016/j.it.2018.06.002 29941203

[160] Reynolds LA Finlay BB Maizels RM. Cohabitation in the Intestine: Interactions Among Helminth Parasites, Bacterial Microbiota, and Host Immunity. J Immunol (2015) 195(9):4059–66. doi: 10.4049/jimmunol.1501432 PMC4617609 26477048

[161] Oliveira-Sequeira TCG David ÉB Ribeiro C Guimarães S Masseno APB Katagiri S. Effect of Bifidobacterium Animalis on Mice Infected With Strongyloides Venezuelensis. Rev Inst Med Trop Sao Paulo (2014) 56(2):105–9. doi: 10.1590/S0036-46652014000200003 PMC4085849 24626410

[162] Dea-Ayuela MA Rama-Iñiguez S Bolás-Fernandez F. Enhanced Susceptibility to Trichuris Muris Infection of B10Br Mice Treated With the Probiotic Lactobacillus Casei. Int Immunopharmacol (2008) 8(1):28–35. doi: 10.1016/j.intimp.2007.10.003 18068097

[163] Entwistle LJ Pelly VS Coomes SM Kannan Y Perez-Lloret J Czieso S. Epithelial-Cell-Derived Phospholipase A(2) Group 1b Is an Endogenous Anthelmintic. Cell Host Microbe (2017) 22(4):484–93.e5. doi: 10.1016/j.chom.2017.09.006 29024642 PMC5644720

[164] Duque-Correa MA Karp NA McCarthy C Forman S Goulding D Sankaranarayanan G. Exclusive Dependence of IL-10rα Signalling on Intestinal Microbiota Homeostasis and Control of Whipworm Infection. PloS Pathog (2019) 15(1):e1007265. doi: 10.1371/journal.ppat.1007265 30640950 PMC6347331

[165] Moyat M Lebon L Perdijk O Wickramasinghe LC Zaiss MM Mosconi I. Microbial Regulation of Intestinal Motility Provides Resistance Against Helminth Infection. Mucosal Immunol (2022). doi: 10.1038/s41385-022-00498-8 PMC9705251 35288644

[166] Viera LQ Moraes-Santos T. Schistosomiasis Mansoni: Evidence for a Milder Response in Germfree Mice. Rev Inst Med Trop Sao Paulo (1987) 29(1):37–42. doi: 10.1590/S0036-46651987000100006 3114864

[167] Holzscheiter M Layland LE Loffredo-Verde E Mair K Vogelmann R Langer R. Lack of Host Gut Microbiota Alters Immune Responses and Intestinal Granuloma Formation During Schistosomiasis. Clin Exp Immunol (2014) 175(2):246–57. doi: 10.1111/cei.12230 PMC3892416 24168057

[168] Hayes KS Bancroft AJ Goldrick M Portsmouth C Roberts IS Grencis RK. Exploitation of the Intestinal Microflora by the Parasitic Nematode Trichuris Muris. Science (2010) 328(5984):1391–4. doi: 10.1126/science.1187703 PMC3428897 20538949

[169] Venzon M Das R Luciano DJ Park HS Kool ET Belasco JG. Microbial Byproducts Determine Reproductive Fitness of Free-Living and Parasitic Nematodes. bioRxiv (2021), 2021.08.02.454806. doi: 10.2139/ssrn.3934611 PMC9187612 35413267

[170] Koyama K. Bacteria-Induced Hatching of Trichuris Muris Eggs Occurs Without Direct Contact Between Eggs and Bacteria. Parasitol Res (2016) 115(1):437–40. doi: 10.1007/s00436-015-4795-2 26481492

[171] White EC Houlden A Bancroft AJ Hayes KS Goldrick M Grencis RK. Manipulation of Host and Parasite Microbiotas: Survival Strategies During Chronic Nematode Infection. Sci Adv (2018) 4(3):eaap7399. doi: 10.1126/sciadv.aap7399 29546242 PMC5851687

[172] Vejzagić N Adelfio R Keiser J Kringel H Thamsborg SM Kapel CMO. Bacteria-Induced Egg Hatching Differs for Trichuris Muris and Trichuris Suis. Parasites Vectors (2015) 8(1):371. doi: 10.1186/s13071-015-0986-z 26174801 PMC4501204

[173] Tenaillon O Skurnik D Picard B Denamur E. The Population Genetics of Commensal Escherichia Coli. Nat Rev Microbiol (2010) 8(3):207–17. doi: 10.1038/nrmicro2298 20157339

[174] Wescott RB. Experimental Nematospiroides Dubius Infection in Germfree and Conventional Mice. Exp Parasitol (1968) 22(2):245–9. doi: 10.1016/0014-4894(68)90099-4 5652501

[175] Chang J Wescott RB. Infectivity, Fecundity, and Survival of Nematospiroides Dubius in Gnotobiotic Mice. Exp Parasitol (1972) 32(3):327–34. doi: 10.1016/0014-4894(72)90060-4 4675136

[176] Wescott RB Todd A. A Comparison of the Development of Nippostrongylus Brasiliensis in Germ-Free and Conventional Mice. J Parasitol (1964) 22(2):138–43. doi: 10.2307/3276048 14125156

[177] Stefanski W Przyjalkowski Z. Effect of Alimentary Tract Microorganisms on the Development of Trichinella Spiralis in Mice. Part I Exp Parasitol (1965) 16(2):167–73. doi: 10.1016/0014-4894(65)90040-8 14281213

[178] Johnson J Reid WM. Ascaridia Galli (Nematoda): Development and Survival in Gnotobiotic Chickens. Exp Parasitol (1973) 33(1):95–9. doi: 10.1016/0014-4894(73)90013-1 4632462

[179] Matijašić M Meštrović T Paljetak HČ Perić M Barešić A Verbanac D. Gut Microbiota Beyond Bacteria-Mycobiome, Virome, Archaeome, and Eukaryotic Parasites in IBD. Int J Mol Sci (2020) 21(8):2668. doi: 10.3390/ijms21082668 PMC7215374 32290414

[180] Maizels RM Nussey DH. Into the Wild: Digging at Immunology's Evolutionary Roots. Nat Immunol (2013) 14(9):879–83. doi: 10.1038/ni.2643 23959175

[181] Scott ME. Heligmosomoides Polygyrus (Nematoda): Susceptible and Resistant Strains of Mice are Indistinguishable Following Natural Infection. Parasitology (1991) 103(3):429–38. doi: 10.1017/S0031182000059953 1780180

[182] Scott ME. High Transmission Rates Restore Expression of Genetically Determined Susceptibility of Mice to Nematode Infections. Parasitology (2006) 132(5):669–79. doi: 10.1017/S0031182005009583 16393368

[183] Sallé G Deiss V Marquis C Tosser-Klopp G Cortet J Serreau D. Genetic × Environment Variation in Sheep Lines Bred for Divergent Resistance to Strongyle Infection. Evol Appl (2021) 14(11):2591–602. doi: 10.1111/eva.13294 PMC8591325 34815741

[184] Srivastava M Misra-Bhattacharya S. Overcoming Drug Resistance for Macro Parasites. Future Microbiol (2015) 10(11):1783–9. doi: 10.2217/fmb.15.73 26517758

[185] Mutombo PN Man NWY Nejsum P Ricketson R Gordon CA Robertson G. Diagnosis and Drug Resistance of Human Soil-Transmitted Helminth Infections: A Public Health Perspective. Adv Parasitol (2019) 104:247–326. doi: 10.1016/bs.apar.2019.02.004 31030770

